# Laser interstitial thermal therapy (LITT) for pediatric low-grade glioma—case presentations and lessons learned

**DOI:** 10.1007/s00381-024-06419-3

**Published:** 2024-05-04

**Authors:** Ido Strauss, Segev Gabay, Jonathan Roth

**Affiliations:** 1 Department of Neurosurgery, Tel Aviv Sourasky Medical Center, Tel Aviv, Israel; 2 Pediatric Neurosurgery and Pediatric Brain Institute, Tel Aviv Sourasky Medical Center, Tel Aviv, Israel; 3 Faculty of Medicine, Tel Aviv University, Tel Aviv, Israel

**Keywords:** Laser interstitial thermal therapy, LITT, pediatric low grade glioma, pLGG

## Abstract

**Background:**

The surgical treatment of brain tumors has developed over time, offering customized strategies for patients and their specific lesions. One of the most recent advances in pediatric neuro-oncological surgery is laser interstitial thermal therapy (LITT). However, its effectiveness and indications are still being evaluated. The aim of this work is to review the current literature on LITT for pediatric low-grade gliomas (pLGG) and evaluate our initial results in this context.

**Methods:**

We retrospectively reviewed our pediatric neurosurgery database for patients who received LITT treatment between November 2019 and December 2023. We collected data on the indications for LITT, technical issues during the procedure, and clinical and radiological follow-up.

**Results:**

Three patients underwent 5 LITT procedures for pLGG. The lesion was thalamo-peduncular in one patient, cingulate in one, and deep parietal in one patient. Two patients had a previous open resection done and were diagnosed with pLGG. One patient underwent a stereotaxic biopsy during the LITT procedure that was non-diagnostic. The same patient underwent a later open resection of the tumor in the cingulate gyrus. There were no surgical complications and all patients were discharged home on the first post-operative day. The follow-up period was between 20 and 40 months. Radiological follow-up showed a progressive reduction of the tumor in patients with LGG.

**Conclusion:**

Laser interstitial thermal therapy is a minimally invasive treatment that shows promise in treating deep-seated pLGG in children. The treatment has demonstrated a reduction in tumor volume, and the positive results continue over time. LITT can be used as an alternative treatment for tumors located in areas that are difficult to access surgically or in cases where other standard treatment options have failed.

## Introduction

MR-guided laser interstitial thermal therapy (LITT) is a minimally invasive surgical technique that uses laser-induced heat to ablate pathological tissue [1]. Over the past decade, LITT has become increasingly accepted as a minimally invasive alternative to open resection for different indications in both adults and pediatrics. The main advantage of LITT is the ability to precisely target deep-seated lesions with minimal disruption to surrounding brain tissue, which is particularly important in areas where the open surgical approach itself is associated with risks of neurological deficits. Although the technique was initially adopted by epilepsy surgeons to ablate epileptic foci (e.g., hypothalamic hamartoma), in recent years, its use has expanded for treating brain tumors [2]. Most of the current literature on LITT for brain tumors focuses on high-grade gliomas and brain metastases in adults, as well as on radiation necrosis following their treatment. The evidence for the use of LITT in low-grade gliomas (LGGs), especially in the pediatric population, is still evolving [3]. In pediatrics, LITT has been used to treat a variety of pathologies, including hypothalamic hamartomas, pilocytic astrocytomas, and subependymal giant cell astrocytomas, among others [2]. The minimally invasive nature of LITT allows for less discomfort and shorter hospital stays which is particularly beneficial for pediatric patients.

LGGs are the most common brain tumor in the pediatric population [4]. Long-term prognosis is generally good, emphasizing the importance of minimizing surgical morbidity and achieving good functional outcomes and quality of life. The current evidence for LITT in pediatric LGGs (pLGG) consists of case series and retrospective studies [3, 5]. These studies suggest that LITT can be safely performed in children with LGGs, with a low incidence of complications, and preservation of neurological function. However, the long-term efficacy of LITT in controlling tumor growth and preventing malignant transformation in LGGs is not yet established. One of the challenges in evaluating the effectiveness of LITT in pediatric LGGs is the heterogeneity of the patient populations and pathologies. Additionally, the natural history of pLGGs, which can be indolent, and the good response to new systemic targeted therapies, make it difficult to assess the impact of LITT on overall survival and progression-free survival.

The expanding use of LITT for pediatric LGG or lesions suspected to be pLGG raises several clinical questions like the optimal timing for recurring tumors, concurrent biopsy for newly diagnosed lesions, trajectory planning, and safety that will be addressed in this manuscript through exemplary clinical case studies.

## Methods

We retrospectively reviewed all pediatric cases who underwent MR-guided LITT at the Tel-Aviv Medical Center between November 2019 and March 2023. Radiological and clinical data was collected. This study was approved by our institutional ethics committee.

### Surgical technique

The technique for Laser interstitial thermal therapy has been extensively described in the literature. The ablation is accomplished by delivering focused laser energy to the target tissue using an optic fiber placed stereotactically inside the tumor. There are currently two FDA-approved LITT systems available in the United States, the Visualase (Medtronic) and NeuroBlate (Monteris). Both systems consist of the same basic components: a laser optic fiber, a laser generator, and a computer imaging workstation that connects to the MRI scanner and controls the system. The key differences between these two systems are the laser wavelength, cooling method, heat production, and distribution pattern. The NeuroBlate system was approved by the FDA in 2009 and uses a 1064-nm diode pulsed laser with a CO2-cooled side-firing probe or diffusing tip probe. The Medtronic Visualase system was approved in 2007 and uses a 980-nm diode continuous laser with a saline-cooled diffusing applicator tip. The Medtronic System is currently the only system that is supported outside the US.

All our LITT procedures were performed using the Medtronic Visualase system. We used a frame-based technique in all our pediatric cases. The patients were placed under general anesthesia and a Leksell G-Frame was attached. Patients were then taken to the CT suite and a stereotactic CT angiography was acquired. Trajectory planning was done by fusing MRI and stereotactic CTA on Brainlab iPlan software and after June 2022 on ROSA software. Stereotactic guidance was performed using the Leksell G-frame Arc until June 2021. We then started using the ROSA robot and registered the frame to the robot using the ROSA localization box. A minimal hair shave was done at the entry site and a stab skin incision was performed. Next, a guided 3.2-mm drill hole in the skull is made, and the dura is opened. At this stage, a stereotactic biopsy can be obtained if necessary using a sedan biopsy needle. A guiding stylet was inserted into the target according to the preplanned trajectory, and the Visualase titanium bolt was fixed to the skull along the planned trajectory. The laser catheter was then inserted into the target through the bolt. We used a 10-mm laser diffusing fiber in all cases. Patients were then transferred to the MRI suite under general anesthesia. Inside the MRI, the laser fiber was connected to the Visualase laser generator. Two orthogonal MR images were selected to monitor the temperature along the trajectory of the catheter. Target thermal protection points were then set to help preserve surrounding critical structures. The tip of the laser fiber can be retracted when needed according to the volume of the lesions and the extension of the thermal damage. Before starting a new ablation, a low-energy test dose is used to verify the location of the diffusing tip.

At the end of the procedure, once the maximum extent of damage was reached, the laser fiber was removed inside the MRI, and post-operative scans were obtained, with diffusion and post-contrast enhancement to evaluate the early effects and possible complications.

Patients were then transferred to the recovery room for awakening. All patients were observed overnight in the pediatric ICU and discharged home the next morning.

## Results

Eight pediatric patients (average age 12.1 years ± SD 2.25 years), six boys, and two girls, underwent 10 LITT procedures, between November 2019 and March 2023. All procedures were performed under general anesthesia. Four patients were operated on for hypothalamic hamartoma and one patient for suspected FCD-causing intractable epilepsy.

Three patients underwent 5 LITT procedures for pLGG, 2 boys, and a girl. All procedures were performed under general anesthesia. Out of the three patients with LGG (Table 1), in one patient, LITT was done as the first surgical option (low-grade left cingulate glial tumor), while in two patients, LITT was done as an additional surgery after a previous open resection (recurrent right thalamo-peduncular pilocytic astrocytoma, and recurrent right parietal oligodendroglioma). In all 5 procedures, a single LITT trajectory was performed with the 10-mm Visualase fiber. There were no morbidity or complications in our cohort, and no wound complications were observed. The follow-up time ranged between 20 and 40 months, (median 30 months, average 28.6 months). Two of the patients with LGG required a second LITT procedure. One of them was planned for a staged procedure (case #2), and the second patient for a distant enlarging lesion (case #3). Volumetric analysis of the tumor (using the Brainlab neurosurgical navigation software; Munich, Germany), before LITT and later and at different time points (Table 2), showed an initial increase in volume immediately post-operatively, then gradually decreasing in size, consistent with the existing literature [6].

**Table 1:** Patient demographic, pathology, and long-term follow-up

**Patient**	**Sex, age (years at 1st surgery)**	**Pathology**	**Location**	**Complications**	**Follow-up time (months)**	**Local\symptoms control**	**Recurrent LITT**
**1**	M, 8.5	Pilocytic astrocytoma	Rt thalamus	No	26	Yes	Yes
**2**	M, 11.4	Low grade oligodendroglioma	Right parietal	No	20	Yes	No
**3**	F, 13.6	Low grade glial tumor	Left cingulate gyrus	No	40	Yes (seizure decreased)	Yes

**Table 2:** Volumetric evaluation of tumor at different times during follow-up MRI

Patient	Surgical procedure	Pre-operation volume (cm^3^)^a^	Post-operation volume	Volume at 1 month	Volume at 3 months	Volume at 6 months	Volume at 12 months
1	#1	15.3	22.2	14.2	13.3	11.8	N\A^b^
	#2	11.8	8.5	7.5	7.1	6	6.5
2	#1	1.02	2	N\A	1.7	1.3	N\A
3	#1	1.09	1.2	N\A	1.3	0.9	0.5
	#2	0.52	1.6	1.3	1.1	0.6	0.4

^a^Cubic centimeters

^b^N\A = not available

## Illustrative cases

### Case 1

**Fig. 1:**
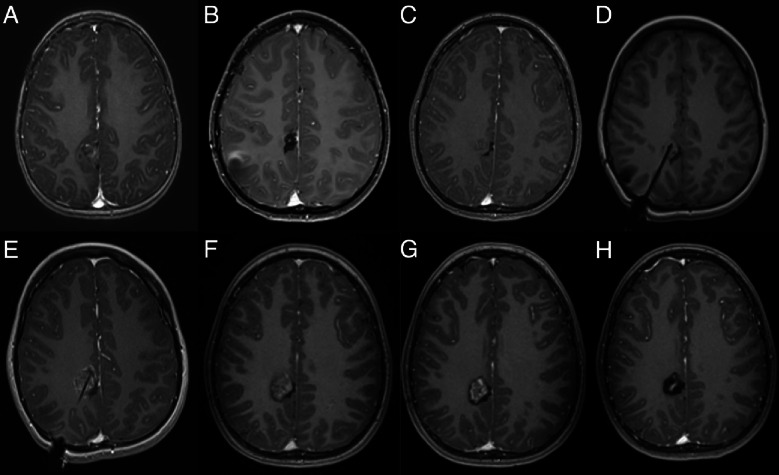
Serial axial T1-enhanced MR images of patient #1. **A** At diagnosis, **B** after resection, **C** tumor recurrence, **D** intraoperative image showing the approach to the tumor, **E** complete ablation of the tumor at the end of surgery, **F**, **G** continues contrast enhancememt 3 and 6 months after surgery, **H** 2.5 years after surgery, demonstrating good local control

An 11-year-old boy presented with a recurrent left parietal deep-seated (post-central) oligodendroglioma. He underwent two open resections at the age of 3 and 4 years, and after the second operation, there was no evidence of residual disease (Fig. 1A, B). The child had mild left hemiparesis. At the age of 9, a small recurrence was observed that continued to grow on serial imaging over a 2-year follow-up period (Fig. 1C). The different treatment options were discussed with the patient’s family including open resection with intraoperative monitoring (IOM). The family did not want to undergo open surgery again, and LITT treatment was agreed on after understanding the risks of motor worsening. DTI was used to delineate the pyramidal tract (PT). A posterior trajectory was chosen so that retraction of the optic fiber would distance the heat from the PT. Immediate post-operative T1-gad scan showed complete ablation of the tumor (Fig. 1E). The patient was discharged home the following day without new neurological deficits. Follow-up imaging showed enhancement that persisted for 6 months after the surgery, with the beginning of cystic changes in the ablated area (Fig. 1G). On longer follow-up imaging, 18 and 30 months after the ablation, there are cystic changes around the tumor bed with minimal enhancement (Fig. 1 H), without evidence of recurrent tumor. The child is doing well.

### Case 2

**Fig. 2:**
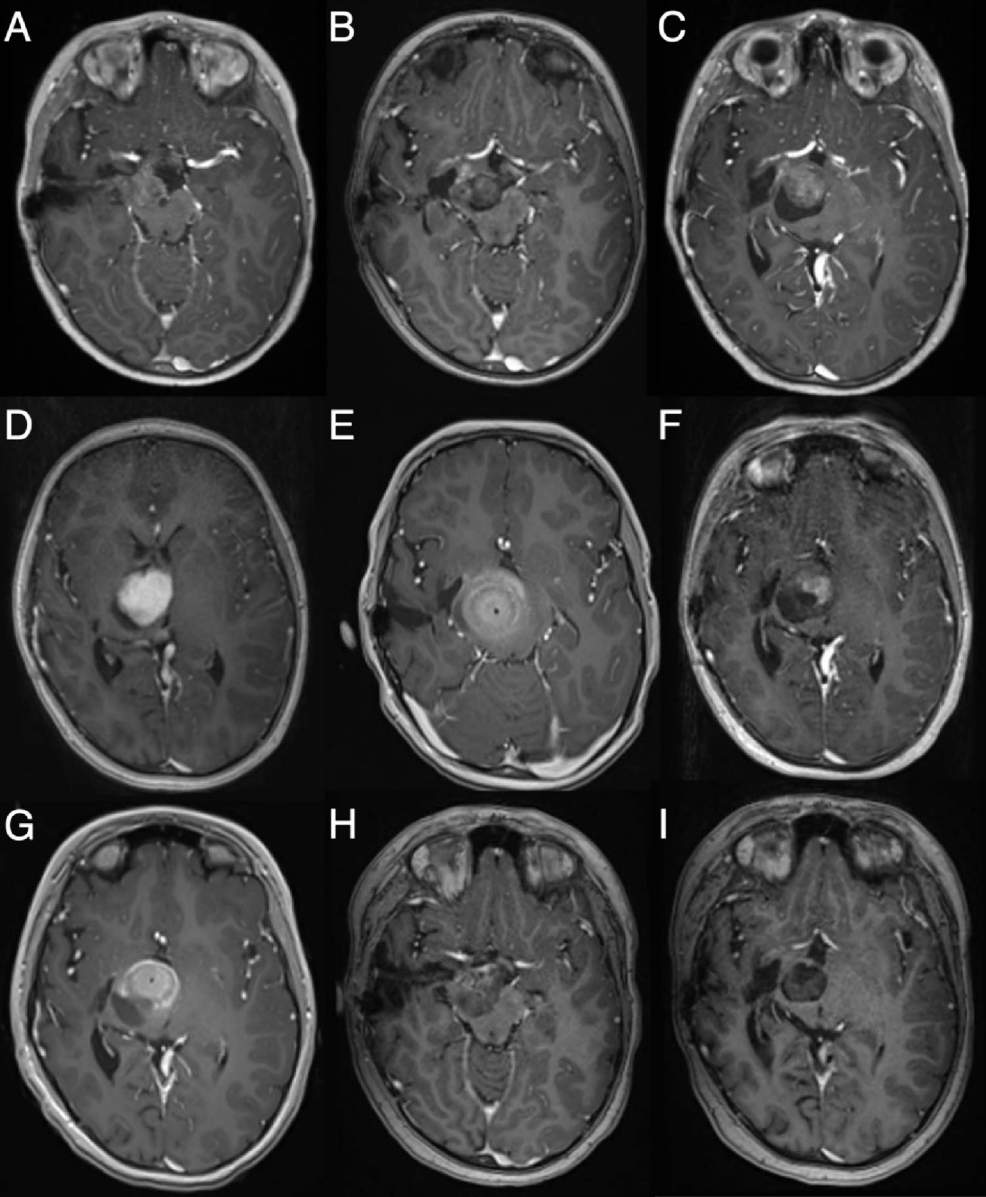
Serial T1-contrast-enhanced axial MR images of case #2. **A** After the resection showing minimal residual tumor, that gradually expanded on follow-up (**B**, **C**). **C** At the first consideration of LITT, **D** continued growth before the first LITT, lesion is > 2 cm in diameter and requires more than one trajectory. **E** At the end of the first ablation. **F** After 6 months, before the second ablation. **G** At the end of the 2nd LITT procedure. **H**, **I** At 2- and 3-year follow-up, respectively

A 7-year-old boy presented to our clinic 3 years after having a partial resection of a right thalamic-peduncular pilocytic astrocytoma (Fig. 2A). The residual tumor continued to grow on serial imaging, and the patient was referred for consideration of LITT (Fig. 2B, C). A significant ablation with LITT was considered possible, but after discussion on the inter-disciplinary tumor board, it was decided to offer the child another line of MEK inhibitors. The patient returned a year later with continued growth of the tumor (Fig. 2D). At this point, it was clear that the tumor could not be ablated using a single trajectory. Due to the size and risk of edema and obstruction of CSF outflow through the third ventricle, we decided to stage the procedure (Fig. 2E). The child recovered well and was discharged the following day, with corticosteroids for 1 week. Six months later after the postoperative changes and edema subsided (Fig. 2F), the residual was targeted with a single trajectory (Fig. 2G). The patient recovered well and was discharged on the following day. On a 3-year follow-up, the tumor has significantly decreased in size, and the residual is not enhancing (Fig. 2I).

### Case 3

**Fig. 3:**
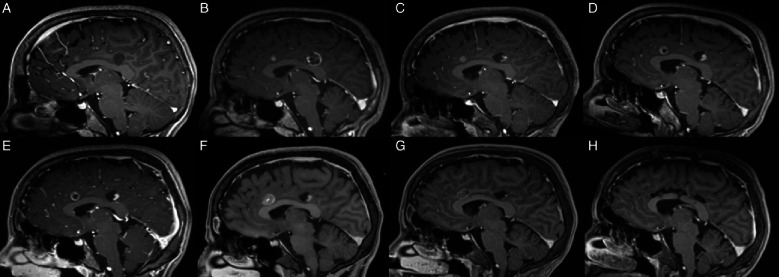
Serial T1-contrast-enhanced sagittal MR images of case #3. **A** At diagnosis, showing the posterior cingulate larger lesion, and a small anterior cingulate enhancing lesion. **B** At the end of the first LITT, the enhancement encompasses the entire posterior lesion. **C**–**E** On follow-up, the anterior cingulate lesion continued to grow. **F** At the end of the second LITT showing good ablation of the anterior cingulate lesion. **G** Six months after the second LITT. **H** After open resection of the cingulate gyrus

**Fig. 4:**
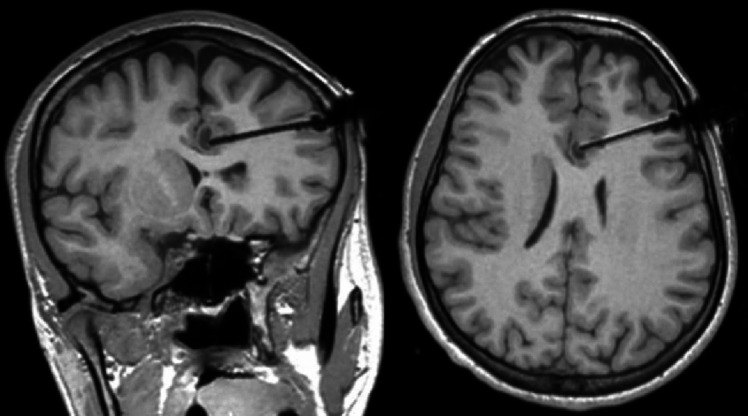
Intraoperative MRI of case #3 demonstrates the laser catheter pushing on the capsule of the lesion without penetrating it

A 13-year-old girl presented due to intractable epilepsy with a multi-focal lesion along the left cingulate gyrus (Fig. 3A). Since the larger posterior lesion was identified as the epileptogenic one, it was decided to treat only that lesion and continue imaging surveillance on the other foci. The main consideration was whether a tissue biopsy was necessary. DTI was used to demonstrate the pyramidal tract, and the distance was considered safe for ablation. After discussing the options with the family, it was decided to perform the ablation without a biopsy, to minimize the risk of bleeding and artifacts that could hinder the ablation [7]. The procedure was successful (Fig. 3B), and the patient was discharged home on the first postoperative day. She remained seizure-free for 2 years. However, the seizures recurred, and on MRI, the anterior cingulate lesion appeared to grow, and a new small lesion appeared in the middle cingulate (Fig. 3C–E). Open resection of the cingulate gyrus was considered, but the patient and family preferred to try another LITT to the anterior lesion combined with a stereotactic biopsy in the same procedure. The stereotactic surgery was uneventful, and after obtaining several samples with a sedan needle, the laser fiber was inserted into the planned target, and the patient was transferred to the MRI suite. On the pre-ablation 3D imaging, the catheter was demonstrated to push the lesion without penetrating it (Fig. 4). We performed the first ablation to test whether the lesion could be penetrated after coagulation. After observing the heat spreading outside the lesion without penetrating it, the whole catheter was deepened by 2–3 mm into the lesion. The ablation of the entire lesion was carried out successfully (Fig. 3F). The child recovered well and was discharged home on POD-1. However, the biopsy was not diagnostic probably because the lesion was not penetrated. On follow-up, the seizures continued, and the middle focus in the cingulate gyrus continued to grow on imaging. Open resection was carried out with gross total resection of the flair changes (Fig. 3H). The child recovered well without new neurological deficits. Pathologic examination demonstrated a low-grade glial tumor with infiltrative and piloid features.

## Discussion

Laser interstitial thermal therapy (LITT) is a neurosurgical technique that is becoming increasingly popular in the pediatric population [2, 3]. PLGGs commonly involve deep and eloquent regions, such as thalamic or thalamo-peduncular, periventricular, and other subcortical locations. The approach to these locations may necessitate crossing functional pathways. The ability to approach these lesions in a minimally destructive manner using an optic fiber to ablate the lesion is appealing. A recent review of LITT in the pediatric population, including 303 pediatric LITT procedures across 35 studies, found that brain tumors were the second most common indication for LITT after epilepsy [2]. LITT was used mainly for deep-seated PLGG, in the periventricular zone or in the thalamus [2]. The largest series published on LITT for pediatric brain tumors, by Arocho-Quinones et al. [8], reported multi-center results on 86 pediatric patients [8], most of them with low-grade (WHO grade I or II) histology [8]. Eight-three percent of the low-grade tumors demonstrated volume reduction at the latest follow-up, suggesting LITT is effective in the management of pediatric low-grade tumors [8].

### Complications

Although LITT is a minimally invasive procedure, it still carries a risk of complications. The exact rate of complications varies between different studies, but one recent analysis of a US national inpatient sample database found a complication rate of 13% and a mortality rate of 2.5% [9]. Similarly, a review of LITT in the pediatric population by Zeller et al. [2] found an overall complication rate of 15.8%, with a 19.1% complication rate in the pediatric tumor subgroup [2]. Arocho-Quinones et al. reported a higher complication rate of 26.7%, most of which were temporary [8]. The mortality rate in their study was 2.3%, with 2 out of 86 cases resulting in death [8]. These complication rates are comparable to the reported complication rate in the adult LITT series [10–12].

Complications during tumor ablation can be categorized into three main groups. The first category includes surgical approach–related complications, such as intracranial hemorrhage or suboptimal catheter placement, which can lead to incomplete tumor ablation. The second category comprises hyperthermia-related neurological injuries that can occur during the ablation process. The extent of these injuries depends on the location of the tumor and relation to eloquent areas and the surgeon’s experience. This category also includes temporary neurological deficits due to post-ablation edema that resolves within a few weeks. The final category includes technical complications related to the laser system [2, 13].

### Extent of resection vs. ablation

The advantages of LITT should be weighed against the ability to achieve complete ablation of the lesion, which depends on several factors, such as the size and shape of the lesion, location, and consistency (heat conductance) (Table 3). It has been repeatedly shown in PLGG that the long-term oncological outcomes are superior with GTR compared to STR [14]. When considering LITT, it is assumed that complete ablation of a tumor is equivalent in terms of local control to resection. A limitation of LITT is that it relies on a statistical thermal damage estimation (TDE) model to predict the area of ablated tissue and intact tumor cells that might be left in the periphery of the ablated zone [15]. Due to the energy absorption/penetration properties of brain tissue at the used laser wavelength (980 nm with Visualase or 1060 nm with Neuroblate), the maximal diameter that can be ablated around a single fiber is up to 18 mm using the Visualase (Medtronic) probe and up to 3 cm using the Neuroblate (Monteris) probe. By using serial ablations along the fiber tract, an elongated cylinder of ablated tissue can be created. Large and irregularly shaped lesions can also be targeted using multiple fibers; however, this adds both risks and cost, which in some countries might be a significant limiting factor [13]. Cystic lesions may be less favorable as the fluid in the cyst serves as a heat sink and dumps the effect of the ablation beyond it.

**Table 3:** Lesion variables and preferred treatment manner

	**Favors LITT**	**Favors open surgery**
Size	< 2 cm	> 2 cm
Location	Deep Not abating eloquent regions	Superficial Near eloquent regions
Consistency	Solid	Cystic
Associated epilepsy		LEAT

*LEAT* Long-term epilepsy–associated tumors

It should also be kept in mind that in contrast to resective surgery, the ablated tissue is left in situ, often transiently expanding in volume after ablation, and might cause increased mass effect and neurological morbidity [16, 17]. This is especially critical near eloquent areas, in closed compartments like the posterior fossa, or near CSF pathways [17, 18]. Staging the ablation for large tumors might lower the risk of edema and hydrocephalus as demonstrated in case #2.

Location near the eloquent cortex or subcortical fibers poses specific challenges to LITT [13, 16]. Careful trajectory planning, taking into account tractography and using low power to understand where and how the heat spreads across the lesion and/or small diffusing tip as necessary might mitigate the risks. However, real-time motor and language mapping using stimulation cannot be performed during the ablation inside the MRI. Awake LITT has been used in adults to mitigate these risks [19]; however, monitoring in the MRI is challenging, and the ablation is stopped only after a mild deficit has already occurred. Open resection, on the other hand, will allow both cortical and subcortical motor mapping, while awake open surgery (even in selected children) will enable the performance of linguistic mapping.

Therefore, choosing appropriate candidates who may benefit from LITT requires careful consideration of the lesion’s specific characteristics and the procedure’s limitations.

### Biopsy

The role of tissue diagnosis is multifactorial, the first is to verify the tumor histology as opposed to other pathologies. Second, beyond histopathological diagnosis, adequate tissue sampling is critical to allow molecular profiling that helps determine prognosis and guide systemic therapies.

For example, BRAF antagonists and MEK inhibitors are the cornerstone biological treatments for specific pLGG [20], while chemotherapeutic regimens are the primary treatment, especially for chiasmatic hypothalamic tumors in infants [21]. While biopsy sampling is possible during the LITT procedure, concerns have been raised by several groups that it may lead to a suboptimal effect of the LITT due to small air bubbles or small bleeds [7, 22]. In addition, tissue sampling is limited and can be non-diagnostic as in case #3, and after ablation is performed there might not be diagnostic tissue to sample from. On the other hand, performing LITT in a separate setting necessitates an additional general anesthesia procedure for the child. In the Arocho-Quinones series, only 74.4% of the cases were proven by biopsy. In 25.6% of the cases, no biopsy was obtained during the LITT procedure, and the diagnosis was presumed based on clinical history and imaging characteristics [8].

In this aspect, the advantage of open resection is that sufficient pathological material is available for histopathology and molecular profiling. Since modern treatments and prognosis are highly dependent on appropriate adjuvant treatment, this should be an important factor when deciding between LITT and open resection in newly diagnosed tumors. This may make any future biopsies of the lesion or its surroundings that were previously treated by LITT to express tissue changes that are not tumoral, potentially leading to a tissue sampling error.

### Epilepsy-associated tumors

Long-term epilepsy–associated tumors (LEAT) are a subgroup of PLGG that causes epilepsy in children [23]. Most tumors are gangliogliomas (GG) and dysembryoplastic neuroepithelial tumors (DNET), which in 50–80% have an associated cortical dysplasia. Thus, treatment often includes not only the lesion (lesionectomy), but also associated dysplasia, often guided by electrocorticography (ECOG). These considerations cannot be addressed using LITT.

### Imaging changes due to BBB opening and therapeutic implications

It is important to be aware of the unique time-dependent imaging changes that occur following LITT. Immediately after the procedure, several tissue zones around the ablation area can be observed (Fig. 2E, G). A central necrosis zone surrounds the optic fiber at the center of the ablation area. This coagulated area where cellular and subcellular membranes are disrupted is characterized on MRI by hyperintensity on T1-weighted images. Around the central necrotic area is a peripheral zone of necrotizing edema, where although irreversible cell damage has occurred, characterized by intra-cellular edema, the initial structural tissue damage is minimal, becoming apparent in the first 48–72 h post-ablation. This area is characterized by hypointensity on T1-weighted images. A 1- to 3-mm enhancing rim, seen in the post-ablation MRI, at the margin of the peripheral zone defines the total volume of thermally induced cell damage.

Beyond that, the damage to the surrounding cells is usually reversible, containing viable cells and reactive perilesional edema. It has been shown that LITT temporarily and locally disrupts the tight junctions that form a major part of the BBB while minimizing collateral damage to the surrounding neuronal tissue [24]. The peak of the increased permeability of the BBB and resulting edema occur 1–2 weeks after laser ablation, extend up to 1–2 cm from the enhancing rim, and usually resolve by 4–6 weeks. The BBB-opening effect has been investigated for its potential to enhance the delivery and response to adjuvant chemotherapy in viable tumor cells that may remain around the ablated area following LITT.

On follow-up imaging, lesions’ volume usually transiently increases in the first 2–4 weeks after the procedure and then gradually decreases, returning to pre-ablation volumes around 3 months after the procedure [25]. This pseudo-progression effect is thought to be mediated by post-ablation inflammatory processes due to thermal necrosis, which, like other ischemic processes, leads to increased vascular permeability and, consequently, increased contrast enhancement [26]. Lesions continue to decrease in volume over time, but it is not uncommon for a small residual enhancing lesion to remain even 6–12 months after the ablation (Fig. 1F, G), without evidence of regrowth. This pseudo-progression might be challenging to differentiate from tumor recurrence and requires serial imaging surveillance.

## Conclusion

LITT is a valid alternative for PLGG. Relatively small, deep, non-cystic lesions are favorable for its use. Concurrent biopsy may be performed, yet it may interfere with the LITT efficacy, and thus, tissue sampling should be minimized. The long-term effects of LITT on PLGG are still unknown, as well as the possible effect on the risk for malignant transformation. Additional, large-scale studies, probably by collaborating with smaller numbers from many centers, should be encouraged. The goals should be to better define the efficacy of LITT on specific pathologies and molecular groups, associated epilepsy, the effect of biopsies on LITT efficacy, and safety using LITT close to various eloquent regions.

## Data Availability

No datasets were generated or analyzed during the current study.
